# Near Real-Time Implementation of An Adaptive Seismocardiography – ECG Multimodal Framework for Cardiac Gating

**DOI:** 10.1109/JTEHM.2019.2923353

**Published:** 2019-09-13

**Authors:** J. Yao, S. Tridandapani, P. T. Bhatti

**Affiliations:** 1 School of Electrical and Computer EngineeringGeorgia Institute of Technology 1372 AtlantaGA30332-0250USA; 2 Department of RadiologyUniversity of Alabama at Birmingham 9968 BirminghamAL35294USA

**Keywords:** Cardiac gating, cardiac quiescence, computed tomography angiography, echocardiography, electrocardiography, multimodal gating, real-time implementation, seismocardiography

## Abstract

*Objective:* Accurate gating for data acquisition of computed tomography (CT) is crucial to obtaining high quality images for diagnosing cardiovascular diseases. To illustrate the feasibility of an optimized cardiac gating strategy, we present a near real-time implementation based on fusing seismocardiography (SCG) and ECG. *Methods*: The implementation was achieved via integrating commercial hardware and software platforms. Testing was performed on five healthy subjects (age: 24–27; m/f: 4/1) and three cardiac patients (age: 41–71; m/f: 2/1), and compared with baseline quiescence derived from echocardiography. *Results*: The average latency introduced by computerized processing was 5.1 ms, well within a 100 ms tolerance bounded by data accumulation time for quiescence prediction. The average prediction error associated with conventional ECG-only versus SCG-ECG-based method over all subjects were 59.58 ms and 27.24 ms, respectively. *Discussion*: The results demonstrate that the multimodal framework can achieve improved quiescence prediction accuracy over the ECG-only-based method in near real-time.

## Graphical abstract

This paper presents the near-realtime implementation of an optimized cardiac gating strategy based on fusing seismocardiography (SCG) and ECG. The results demonstrate that the multimodal framework can achieve improved quiescence prediction accuracy over the ECG-only-based method in near real-time.

## I. Introduction and Clinical Need

Cardiovascular disease (CVD) is the leading cause of death globally. About eight million Americans suffer from CVDs with 2,400 deaths daily [1]. The gold standard for evaluating CVDs, catheter coronary angiography, is invasive and expensive. An alternative technique, computed tomography angiography (CTA), is less invasive, relatively inexpensive and faster [2]. However, this emerging diagnostic tool suffers from limited temporal resolution. Due to the heart motion, artifacts present and compromise the diagnostic quality. To reduce cardiac motion artifacts and minimize radiation exposure, cardiac CTA data acquisition requires triggering during cardiac quiescence, i.e., when cardiac motion is minimal within the cardiac cycle.

This study builds on our earlier work in developing SCG- and SCG-ECG-based quiescence prediction methods (a.k.a. weighted fusion, or WF [3], [4]). Both methods demonstrate improved quiescence prediction accuracy over the sub-optimal ECG-only-based method during off-line testing, with the multimodal SCG-ECG method predicting quiescence that is temporally closer to the gold-standard of ultrasound-based prediction [5]. Furthermore, the diagnostic quality of CTA reconstructed images was evaluated, and improvement was observed. Thus, the multimodal gating strategy with the inclusion of SCG is promising in improving diagnostic quality, while reducing radiation exposure (from 12 mSv to 4 mSv) to cardiac patients [6].

The utility of multimodal gating has been demonstrated by other groups. In 2004, General Electric Medical Systems (Boston, MA, USA) was awarded a patent regarding the invention of mechanical CTA gating [7]. However, the proposed schematic design has yet to be implemented. In 2008, the real-time DTU200/300 dual channel MRI triggering and gating system (BIOPAC Systems, Goleta, CA, USA) [8] was released with the purpose of improving tumor and/or lesion delineation on abdominal imaging by minimizing respiratory motion artifacts [9]. Although the respiratory cycle is of lower frequency compared with cardiac cycles, this real-time implementation demonstrated positive outcomes for dual-gating in cardiac CTA. More recently, two approaches of multimodal gating for positron emission tomography (PET) imaging were developed, both of which demonstrated the potential of cardiac-motion-based signal in cardiac PET imaging [10], [11]. However, the feasibility of these dual-sensor approaches in real-time cardiac gating applications has not been established.

**FIGURE 1.**
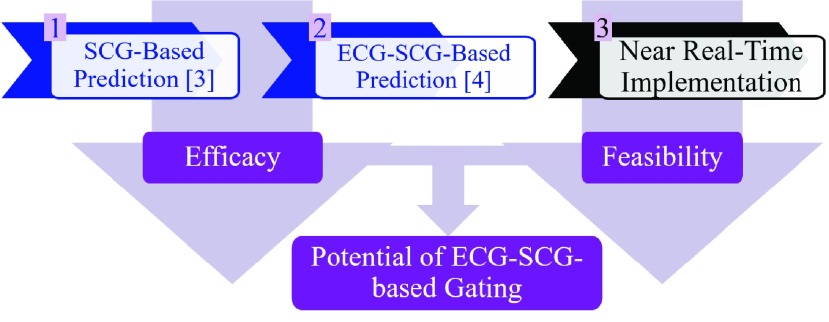
Overall strategy of the multimodal gating method. The SCG- [3] and ECG-SCG-based [4] prediction methods were developed to demonstrate the efficacy of cardiac-motion-based signal in quiescence prediction. The near real-time implementation evaluates the feasibility of the developed methods. The ultimate goal is investigating the potential of multimodal gating strategy in clinical practice.

As a step forward, we implement the multimodal gating strategy for cardiac CTA in real-time to validate its feasibility in clinical practice. Figure 1 illustrates the overall strategy of multimodal gating. A detailed description of the underlying prediction method may be found in [3], [4]. The most notable difference in this communications paper is the translation of an offline system to a near real-time system realized via readily available commercial hardware and software described in the Methods section.

## II. Methods

**FIGURE 2.**
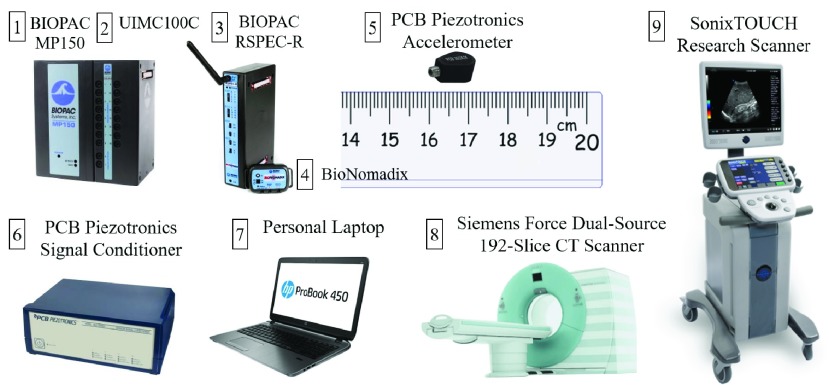
Devices used in this work. The processing is based on MATLAB 2017 running on a Hewlett-Packard computer with an Intel 4-core. The technical details of the individual device components can be found in Table 1 of the supplementary document.

Figure 2 summarizes the commercial hardware and software platforms integrated to develop the near real-time gating platform. The MP150 (BIOPAC Systems) enables synchronized data acquisition and real-time analysis of the ECG and SCG signals. Upon receipt by the MP150, analog signals were individually filtered/conditioned by their corresponding biopotential amplifier modules (SCG: BIOPAC UIMC100C and PCB Piezotronics signal conditioner; ECG: BIOPAC RSPEC-R). Both analog signals were then sampled by an analog-to-digital converter at a rate of 200 Hz. The BIOPAC hardware application programming interface (BHAPI) enabled interaction between MP150, and third-party software programs, i.e. MATLAB (MathWorks Inc, Natick, MA, USA), for data processing. Specifically, BHAPI allows for streaming data transfer and programming interface via a dynamic link library.

**FIGURE 3.**
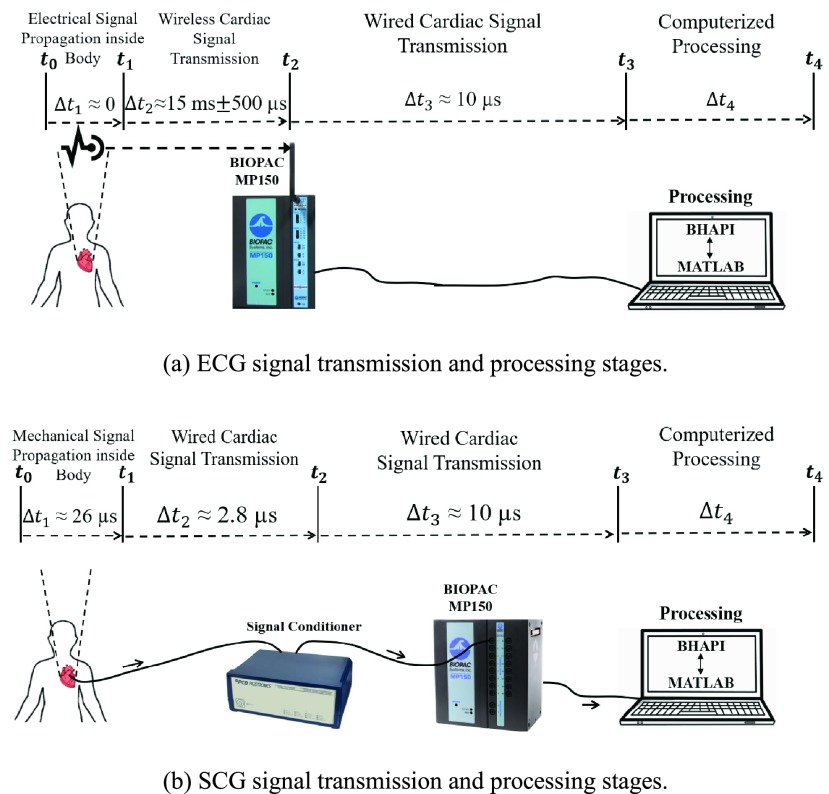
Breakdown of stages of (a) ECG and (b) SCG signals transmission and processing. The latency introduced by each stage i is denoted as $\Delta t_{i}$ for i = 1, 2, 3, 4.

Latency is an important aspect of the real-time implementation and is crucial for quiescence predictions. The true quiescence can be missed if the total latency exceeds the true duration prior to CTA data acquisition for a cardiac cycle, i.e. missed cardiac cycles due to quiescence occurring prior to the predicted period. The time latency was quantified by evaluating the latency introduced by each individual stage of signal transmission. A breakdown of the cardiac signal transmission latencies for ECG and SCG are presented in Fig. 3.

Due to different device configurations, the transmission duration of ECG and SCG signals can vary for the same stage. In transmitting the ECG signal to the MP150, the wireless transceiver introduces a 15 ms biased delay and $500~\mu \text{s}$ unbiased delay. In contrast, the wired signal transmission duration of the SCG signal to the signal conditioner is negligible. However, the SCG signal conditioner introduces $\sim 2.8~\mu \text{s}$ of delay for the amplification operation. The multiplexer switching between two signal channels during stage $t_{2}-t_{3}$ causes a $5~\mu \text{s}$ delay and the ADC causes another $5~\mu \text{s}$ delay [12], resulting in a total of $10~\mu \text{s}$ of delay. In summary, the total latency introduced by the signal transmission and computerized processing is $\Delta T = \sum \nolimits _{i=1}^{4}\,\,t_{i} $. Specifically, the latency is $\Delta T_{\mathrm {ecg}} = 15.51$ ms $+ \mathrm {\Delta }t_{4}$ for the ECG signal, and $\Delta T_{\mathrm {scg}} = 38.8\,\,\mu \text{s}\,\,+ \Delta t_{4}$ for the SCG signal. The generation of WF-based prediction involves both ECG and SCG signals, thus the total latency for WF-based method is $\Delta T_{\mathrm {wf}}=\max \left ({\Delta T_{\mathrm {ecg}},\Delta T_{\mathrm {scg}} }\right)=15.51 \mathrm {ms} + \Delta t_{4}$. For a normal subject with an average heartbeat of 75 bpm (800 ms), 15.51 ms translates to only 2% of a regular heartbeat, thus leaving sufficient margin before quiescence occurs. It was found that on average, the center of systolic and diastolic quiescent periods was at 29% and 76% for healthy subjects, and 33% and 79% for subjects with CVDs [13].

Concerning the computerized processing stage, the quiescence prediction was made on a beat-by-beat basis in a near real-time manner, using ECG-only- and SCG-ECG-based methods, respectively. A high-level block diagram of the computerized processing modules is presented in Fig. 2 of the supplementary document. The quiescence derived from the subject-specific B-mode echocardiography (Sonix RP Scanner, BK Ultrasound, Richmond, BC, Canada) was used as the baseline or gold-standard when comparing the performance of the two methods. Frame-based processing was employed during real-time processing. The size of the processing frame was 97, and the sliding offset was 20 data samples.

## III. Results

The latency and quiescence prediction accuracy were assessed on five healthy subjects (age: 24–27; m/f: 4/1) and three cardiac patients (age: 41–71; m/f: 2/1). The estimated total latency of 5.10 ms introduced by computerized processing is within a 100 ms tolerance, which is the duration of accumulating 20 incoming samples at a sampling rate of 200 Hz for processing. The latencies of individual computerized processing modules are measured and listed in Tables 2 and 3 of the supplementary document.

Table 1 reports the average quiescence prediction error of ECG- and ECG-SCG-based methods for each individual as well as the missing count for the WF-based prediction, in order of increasing heart rate within each cohort (healthy, cardiac). The missing count is the number of cardiac cycles that did not have predictions from the SCG-ECG-based method. Overall, real-time WF-based prediction was feasible and was more accurate than the ECG-only-based prediction.

**TABLE 1:** Average Quiescence Prediction Error

Healthy Subjects					
Subject	HR (bpm)	HRV (ms)	MN/TN^†^	$\bar {E}_{\mathrm {ecg}} $ (ms)	$\bar {E}_{\mathrm {wf}}$ (ms)
H1	53	28	20/286	55.49	22.21
H2	58	45	12/305	57.14	17.56
H3	72	50	25/311	43.66	29.38
H4	75	50	19/275	48.07	22.47
H5	76	32	23/298	66.43	32.11
Mean	69	41	20/295	54.15	24.75
Std	9.4	10.3	5/15	19.56	11.86
Cardiac Patients					
P1	63	102	20/286	58.02	30.04
P2	73	57	25/301	79.81	35.44
P3	80	60	27/300	68.03	28.73
Mean	72	73	24/296	68.62	31.40
Std	8.5	25.1	4/8	17.32	11.51

^†^ MN: Number of missed cardiac cycles due to failure in identifying the SCG feature using the WF-based method or due to quiescence occurring prior to the predicted period. TN: Total number of cardiac cycles.

## IV. Discussion

This work demonstrated the feasibility of a near real-time ECG-SCG multimodal framework for cardiac CTA gating, and motivates further development of a multimodal gating method for clinical application.

One limitation of this work is the use of patient-specific echocardiography as the baseline for coronary vessel motion. It is undesirable to obtain CTA data as the baseline over a large number of cardiac cycles on human subjects due to the attendant excessive radiation. Animal studies may be an acceptable alternative. Another limitation is the small sample size, subject population and narrow demographics.

## V. Future Directions and Potential Clinical Impact

Future work can focus on developing an independent device dedicated to near real-time prediction. A schematic layout of an independent application-specific device is presented in Fig. 4 of the supplementary document. In addition, improving the computerized processing via optimizing the execution time of processing modules from aspects of the algorithms, data structure, input, and programming language may be investigated.

The promising results of this work reinforce the prospect of multimodal gating applications to the clinical setting. The next generation of cardiac imaging machines, including CTA and MRI, can potentially be triggered more accurately in real-time by using a multimodal framework rather than ECG alone.
